# Efficacy and safety of left atrial appendage closure compared with oral anticoagulation in atrial fibrillation: a meta-analysis of randomized controlled trials and propensity-matched studies

**DOI:** 10.3389/fcvm.2023.1212161

**Published:** 2023-09-27

**Authors:** Luca Franchin, Francesco Piroli, Pierluigi Demola, Francesca Mantovani, Mario Iannaccone, Roberto Manfredi, Fabrizio D’Ascenzo, Federico Fortuni, Fabrizio Ugo, Francesco Meucci, Alessandro Navazio, Giacomo Boccuzzi

**Affiliations:** ^1^ Department of Cardiology, San Giovanni Bosco Hospital, Turin, Italy; ^2^ Department of Cardiology, Azienda Sanitaria Universitaria Friuli Centrale, Udine, Italy; ^3^ Cardiology Unit, Azienda USL-IRCCS di Reggio Emilia, Reggio Emilia, Italy; ^4^ Cardiology and Arrhythmology Clinic, University Hospital “Ospedali Riuniti”, Ancona, Italy; ^5^ Division of Cardiology, Department of Medicine, A.O.U Città Della Salute e Della Scienza, Turin, Italy; ^6^ Department of Cardiology, San Giovanni Battista Hospital, Foligno, Italy; ^7^ Department of Cardiology, Leiden University Medical Center, Leiden, Netherlands; ^8^ Structural Interventional Cardiology, Department of Clinical and Experimental Medicine, Careggi University Hospital, Florence, Italy; ^9^ Division of Cardiology, Sant’Andrea Hospital, Vercelli, Italy

**Keywords:** atrial fibrillation, left atrial appendage closure, oral anticoagulation, meta-analysis, cardiovascular intervention

## Abstract

**Backgrounds:**

Two recent randomized controlled trials (RCTs), the PROTECT-AF and the PREVAIL, showed that in atrial fibrillation (AF) patients, left atrial appendage closure (LAAC) is comparable to oral anticoagulants (OAC) in the prevention of stroke and could also possibly reduce mortality. Nevertheless, this net clinical benefit was not confirmed in the most recent RCT comparing LAAC vs. OAC, the PRAGUE-17 trial.

**Aim:**

aim of the present study was to evaluate the efficacy and safety of LAAC compared with OAC among available high-quality studies.

**Methods:**

A systematic search of electronic databases (Medline, Scopus, Embase and the Cochrane Library) was performed to identify eligible RCTs and observational studies with propensity score matching (PSM) analysis. PRISMA guidelines were used for abstracting data and assessing data quality and validity. Outcomes of interest were the occurrence of cardiovascular death (CVD), all-cause death, all-type stroke, and major bleedings.

**Results:**

A total of 3 RCTs and 7 PMS studies involving 25,700 patients were identified. 12,961 patients received LAAC while 12,739 received OAC therapy. After a median follow-up of 2.6 years (IQR 2–4.4), patients who received LAAC had lower risk of CVD (RR = 0.62; 95%CI, 0.51–0.74, *I*^2 ^= 0%), all-cause death (RR = 0.67; 95% CI, 0.57–0.78, *I*^2^ 68%) and major bleedings (RR = 0.68; 95%CI, 0.48–0.95 *I*^2 ^= 87%) compared with patients on OAC. No difference was found between the two groups regarding strokes incidence (RR = 0.94; 95% CI, 0.77–1.15, *I*^2 ^= 0%).

**Conclusions:**

According to this meta-analysis, LAAC has comparable efficacy in the prevention of stroke compared with OAC and a reduced risk of major bleedings, all-cause death and CVD that may be even larger with longer follow-up.

**Systematic Review Registration:**

https://www.crd.york.ac.uk/prospero/display_record.php?RecordID=269768, identifier CRD42021269768.

## Introduction

The therapeutic mainstay for stroke prophylaxis in non-valvular atrial fibrillation (AF) is represented by oral anticoagulation, historically with vitamin K antagonist (VKA) and nowadays with direct oral anticoagulants (DOACs) due to their safer profile risk. Several studies have shown that the left atrial appendage is the most common site of intracardiac thrombi (1, 2) even in patients with sinus rhythm (3). In this regard, transcatheter left atrial appendage closure (LAAC) has been growingly considered as a therapeutic alternative in high bleeding risk patients with AF not suitable for lifelong anticoagulation therapy (4, 5). In the last decade, two randomized controlled trials (RCTs), the Percutaneous left atrial appendage closure vs. warfarin for atrial fibrillation a randomized clinical trial (PROTECT-AF) and the Percutaneous closure of the left atrial appendage vs. warfarin therapy for prevention of stroke in patients with atrial fibrillation: a randomised non-inferiority trial (PREVAIL) (6, 7) demonstrated the non-inferiority of this treatment compared with oral anticoagulants (OAC) in the prevention of stroke. The long-term follow-up data of the PROTECT-AF trial showed even a statistically significant improvement in the reduction of cardiovascular death (CVD) rate in the LAAC arm compared with OAC (6). Nevertheless, this net clinical benefit was not confirmed in the most recent RCT comparing these two strategies, the Left Atrial Appendage Closure vs. Novel Anticoagulation Agents in High-Risk Atrial Fibrillation Patients (PRAGUE-17) trial (8). Giving these conflicting findings, the role of LAAC in patients with AF is still a matter of debate. Accordingly, the aim of this meta-analysis was to compare the safety and efficacy of LAAC vs. OAC for the prevention of cardiovascular events in patients with AF.

## Methods

**Figure 1:**
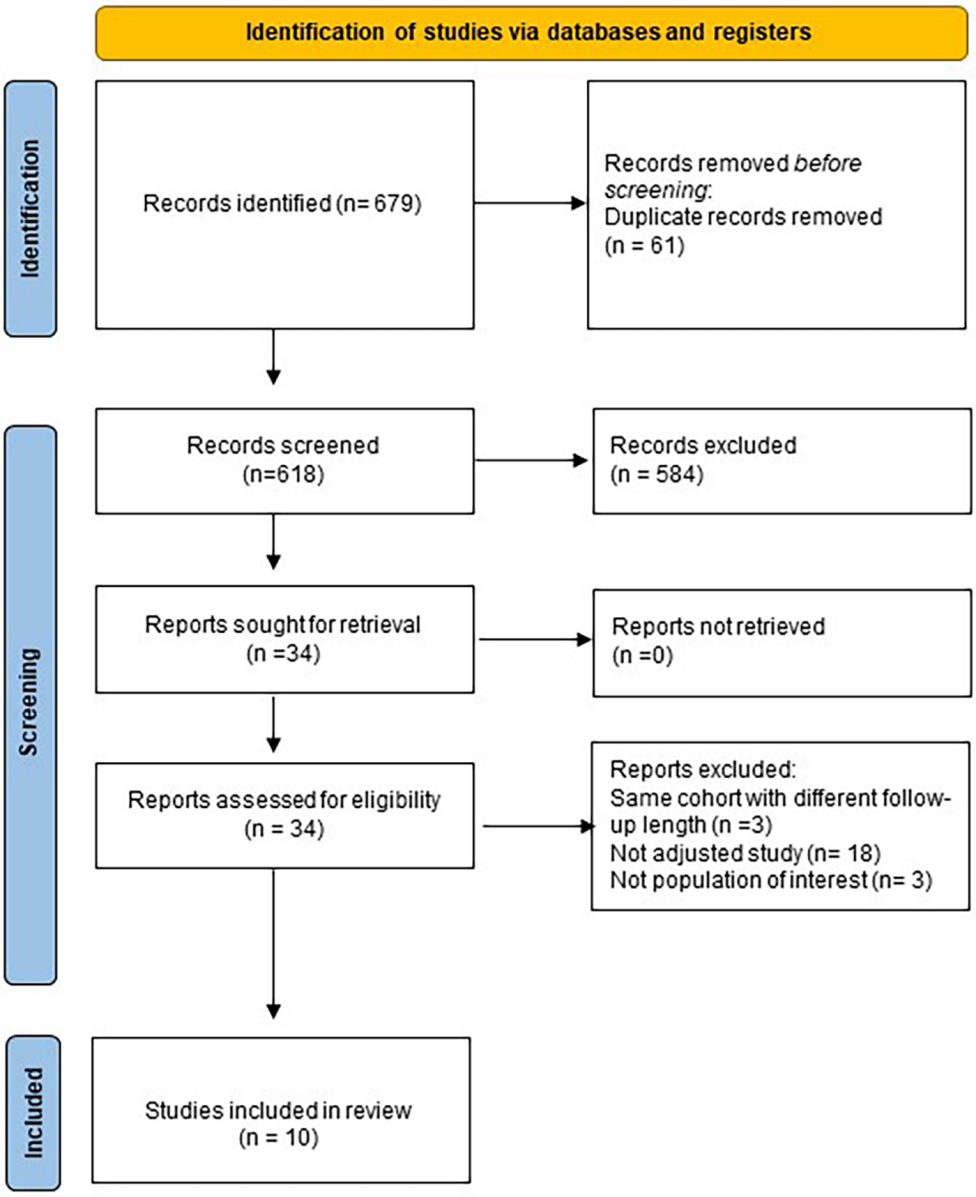
Flowchart of study selection process.

A systematic search of electronic databases (Medline, Scopus, Embase and The Cochrane Library) was performed to identify eligible RCTs and observational studies with propensity score matching (PSM) analysis from database inception to February 2023. The query used was: “((OAC)or(NOAC)or(DOAC)or(warfarin)or(rivaroxaban)or(dabigatran)or(edoxaban)or(apixaban))AND((appendage occlusion)or(watchman)or(appendage closure)or(amulet)or(lambre))”. The search was limited to English papers. Reference letters, reviews, meta-analyses and editorials were also checked to identify potentially eligible studies. The process was performed according to the Preferred Reporting Items for Systematic Reviews and Meta-Analyses (PRISMA) and Meta-analysis Of Observational Studies in Epidemiology (MOOSE) statement (9–11). The original study protocol was registered on the PROSPERO platform (CRD42021269768). To be eligible for inclusion, studies had to report baseline characteristics of patients, procedural features and at least one of the outcomes of interest, only studies with a minimum follow-up of twelve months were appraised. When different studies covered the same population, only the article with the longest follow-up was considered. Two independent investigators (FP, LF) reviewed all titles and abstracts and selected the potentially eligible papers. For each eligible study, full texts were thoroughly examined to assess inclusion and exclusion criteria and discrepancies were resolved by consensus. Flowchart of the study selection process may be found in Figure 1.

The co-primary endpoints of interest were the rate of all-cause death, CVD, all-type stroke and major bleedings at the longest follow-up. The secondary endpoints were the rate of ischemic and hemorrhagic stroke at the longest follow-up. Statistical pooling was performed according to a random-effect model with Mantel-Haenszel weighting method, computing risk ratios with 95% confidence intervals (CI), using Review Manager 5.4.1 (The Cochrane Collaboration, the Nordic Cochrane Centre, Copenhagen, Denmark). A two-sided *p*-value <0.05 was considered significant. Risk Ratio (RR) with 95% confidence intervals (CIs) were reported. Heterogeneity was assessed using Cochrane Q test and *I*^2^ index, which describes the percentage of total variation across the studies that is due to heterogeneity rather than chance (12). *I*^2^ values of 25%, 50%, and 75% represented small, moderate and large amounts of heterogeneity, respectively. A leave-one-out sensitivity analysis and a subgroup analysis based of study design (RCTs vs. PSM) were performed to evaluate whether the results were largely affected by single studies or type of the study. Meta-regression analysis was performed to assess the impact of main baseline features on stroke and major bleeding risk with Comprehensive Meta-analysis software (Biostat, Inc., Englewood, U.S.A).

The presence of eventual publication bias was assessed by funnel plot visual inspection. Quality of study assessment was performed by two independent investigators (RM, FP) by means of the ROB2 tool for RCTs (13) and ROBINS-I tool (14) conflicts were resolved by consensus.

## Results

Overall, 679 titles and abstracts were identified through database searching from inception to February 28th, 2023; after exclusion according to pre-specified criteria, 10 studies were included in the present analysis (Figure 1).

**Figure 2:**
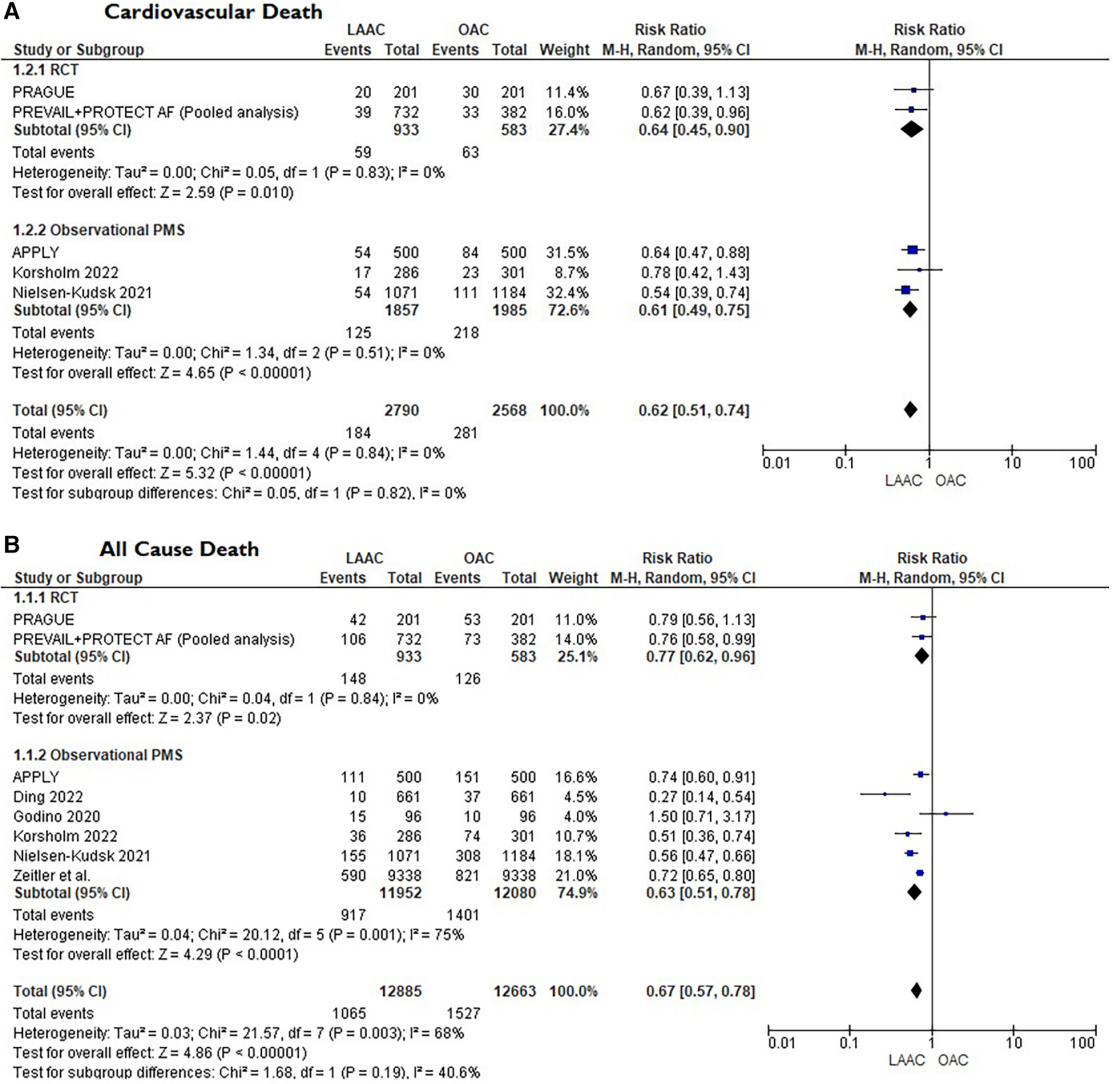
(**A**) Forest plot reporting risk ratios for cardiovascular mortality in LAAC patients compared with patients on OAC. (**B**) Forest plot reporting risk ratios for all-cause death in LAAC patients compared with patients on OAC. (**C**) Forest plot reporting risk ratios for major bleedings in LAAC patients compared with patients on OAC. (**D**) Forest plot reporting risk ratios for stroke in LAAC patients compared with patients on OAC.

**Figure 3:**
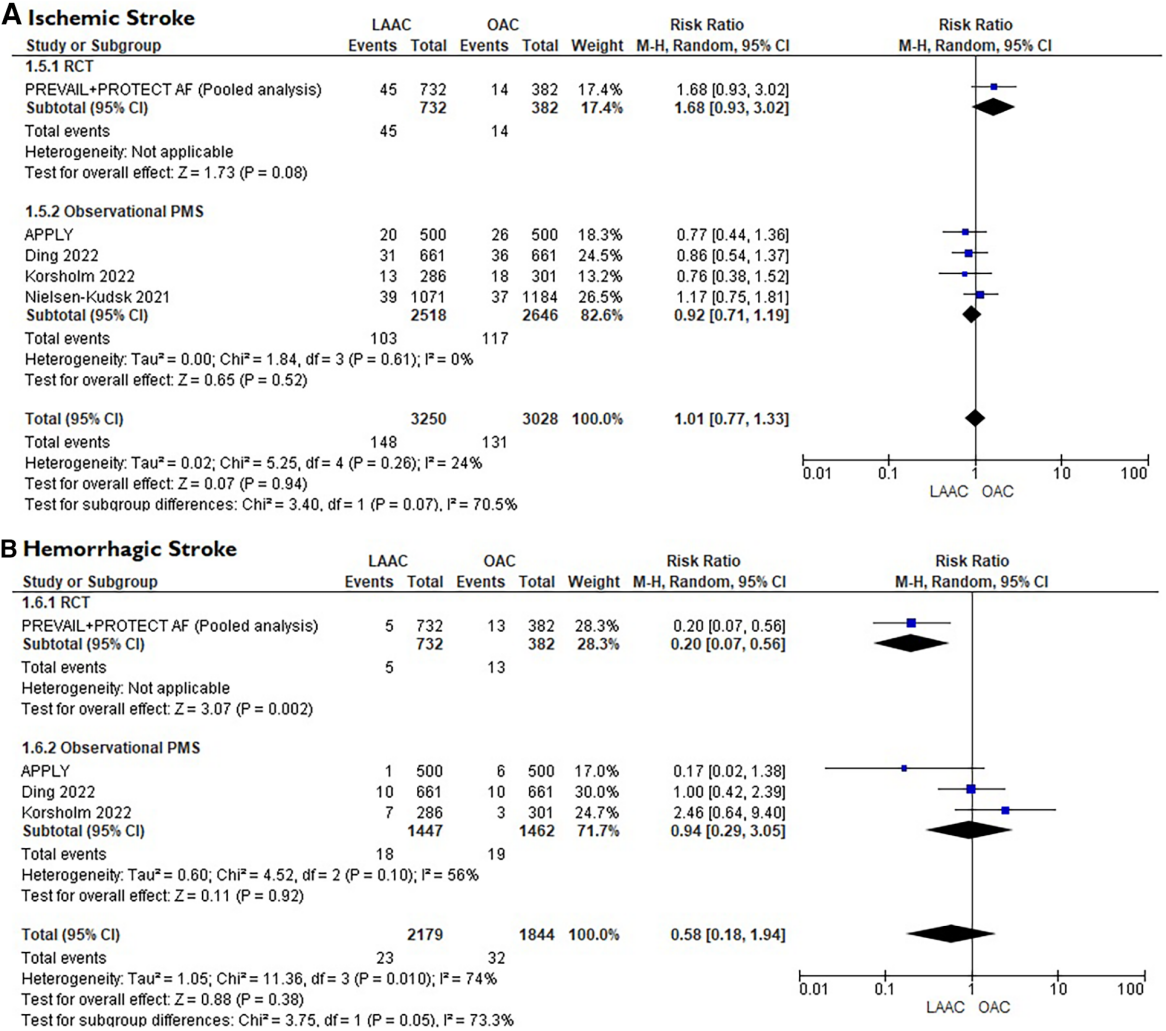
(**A)** Forest plot reporting risk ratios for ischemic stroke in patients LAAC patients compared with patients on OAC. (**B**) Forest plot reporting risk ratios for hemorrhagic stroke in patients LAAC patients compared with patients on OAC.

A total of 10 studies [3 RCTs (6, 8, 15) and 7 PSM studies 16–22] involving 25,700 patients were identified. 12,961 patients received LAAC while 12,739 received OAC therapy. The patients had a median age of 74 years (IQR 71–75) and 34% were women. The median CHA_2_DS_2_-VASc and HASBLED were 4.3 and 3.3 respectively. The baseline and main characteristics of the studies and relative populations are reported in Tables 1, 2. After a median follow-up of 2.6 years (IQR 2–4.5) patients who received LAAC had a lower risk of CVD (RR = 0.62; 95% CI, 0.51–0.74, *I*^2 ^= 0%, Figure 2A), all cause death (RR 0.67; 95% CI, 0.57–0.78, *I*^2 ^= 68%, Figure 2B) and major bleedings (RR = 0.68; 95%CI, 0.48–0.95 *I*^2 ^= 87%, Figure 2C) compared with patients on OAC. Regarding the occurrence of stroke, no differences were found between the two groups (RR = 0.94; 95%CI, 0.76–1.16, *I*^2 ^= 0%, Figure 2D), regardless of the type of stroke considered (RR 1.01;95%CI, 0.76–1.35, *I*^2 ^= 24% for ischemic stroke) (Figure 3A) (RR 0.58;95%CI, 0.17–1.96, *I*^2 ^= 74% for hemorrhagic stroke) (Figure 3B). Results remained consistent at the leave-one-out analysis for all the co-primary outcomes. Subgroup analysis for study design were consistent for all the co-primary outcomes except for major bleedings where the protective role of LAAC compared with OAC reached the statistical significance only in PSM subgroup analysis.

**Table 1:** Baseline characteristics.

Study name	Total patients	LAAC patients	OAC patients	Age (%)	Female (%)	CHA_2_DS_2_VASC	HASBLED	OAC Patients	
								DOAC	VKA
PRAGUE 17	402	201	201	73.3	34.3	4,7	3.1	100%	0%
PREVAIL	407	269	138	74.5	28.9	3.9	–	0	100%
PROTECT AF	707	463	244	72.2	29.8	–	–	0	100%
APPLY	1,000	500	500	74	31	4.3	3	53.6%	25.2%
Nielsen-Kudsk 2021	2,255	1,071	1,184	75.1	37,2	4.3	3.4	100%	0
Godino 2020	192	96	96	74.5	33	4.3	3.5	100%	0
Ding 2022	1,322	661	661	69.5	34	–	–	100%	0
Korsholm 2022	587	286	301	76.2	33.2	5.3	4	100%	0
Falasconi 2021	52	26	26	70.8	26.9	4.3	3	100%	0
Zeitler 2023	18,776	9,388	9,388	75.5	48	5	–	52%	48%

DOAC, direct oral anticoagulants; FU, follow-up; LAAC, left atrial appendage closure; OAC, oral anticoagulation; VKA, vitamin K antagonist.

**Table 2:** Main features of included studies.

Name	Design	Inclusion criteria	Exclusion criteria	Study enrolment	Enrolled patients	Arms	Primary end points	Follow up (years)
PRAGUE 17	Multicenter, open-label, RCT	Bleeding requiring intervention/ hospitalization, cardioembolic event while taking OAC; moderate to high risk CHA2DS2-VASc or HAS-BLED >3	Mechanical valve prosthesis, mitral stenosis, comorbidities other than AF mandating anticoagulation, mobile aortic plaque, symptomatic carotid disease, bleeding within 30 days	Oct 2015 -Jan 2019	402	LAAC (*n* = 201) DOACs (*n* = 201)	Composite of cardioembolic events (stroke, TIA, or systemic embolism), CVD, clinically relevant bleeding, or procedure-/device- related complications (LAAC group only)	3,5
PREVAIL	Multicenter, RCT	Patients with non valvular AF (paroxysmal, persistent, or permanent) and a CHADS2 score >2	OAC for reasons other than AF, contraindication to VKA or aspirin, previous stroke/TIA within 90 days of enrollment, symptomatic carotid disease	June 2008–October 2012	407	LAAC (*n* = 269) VKA (*n* = 138)	Composite of hemorrhagic or ischemic stroke, SE, and cardiovascular/unexplained death	5
PROTECT AF	Multicenter, RCT	>18 years with non-valvular AF with CHADS2 risk score of 1 or more	Contraindications to warfarin, comorbidities other than AF requiring VKA, LAA thrombus, mobile aortic atheroma, symptomatic carotid disease	February 2005–June 2009	707	LAAC (*n* = 463) VKA (*n* = 244)	Composite of stroke (including ischaemic or haemorrhagic stroke), cardiovascular or unexplained death, or systemic embolism	5
APPLY	Dual-center observational retrospective study with a PSM 1:1 ratio	Consecutive patients who underwent LAAC with dedicated AMPLATZER occluders	Overt infection, endocarditis, pregnancy, intracardiac thrombus, and reasons for OAC other than AF, malignant conditions	September 2016–December 2018	1000	LAAC (*n* = 500) VKA/DOACs (*n* = 500)	Composite of stroke, systemic embolism and cardiovascular/unexplained death	2,7
Nielsen-Kudsk 2021	Multicenter, observational prospective compared with a PSM control cohort	Nonvalvular AF enrolled in the Amulet Observational Study with successful LAAC	–	June 2015 –September 2016	2,255	LAAC (*n* = 1,071) DOACs (*n* = 1,184)	Composite of ischemic stroke, major bleeding or all-cause mortality.	2
Godino 2020	Single-center, observational prospective with a PSM 1:1 ratio	Consecutive patients with non-valvular AF who underwent successful percutaneous LAAC	Valvular AF	July 2009–December 2016	382 unmatched (192 PSM)	LAAC (*n* = 96) DOACs (*n* = 96)	Composite of ischemic stroke, TIA, SE, acute myocardial infarction	2
Ding 2022	Multicenter, observational retrospective study with a PSM 1:1 ratio	Aged over 18 years received LAAC or NOAC therapy	Rheumatic heart disease and acute rheumatic fever	December 2010–January 2019	1,08,697 unmatched (1322 PSM)	LAAC (*n* = 661) DOACs (*n* = 661)	All-cause mortality, Composite of thrombotic and thromboembolic events, ischaemic stroke or TIA, venous thromboembolism and intracranial haemorrhage.	2
Korsholm 2022	Multicenter, observational retrospective study with a PSM 1:2 ratio	AF patients enrolled in the Amulet Observational Study having history of ischemic stroke and technically successful treatment by LAAC	Intracardiac thrombus, active infection or endocarditis, LAA anatomy not accommodating a device according to guidelines	2015–2016	587	LAAC (*n* = 286) DOACs (*n* = 301)	Composite of ischemic stroke, major bleeding or all-cause mortality	2
Falasconi 2021	Dual-center, observational retrospective study with a PSM 1:1 ratio	Non-valvular AF patients experienced CE despite OAC	Symptomatic carotid disease	September 2012–July 2019	52	LAAC (*n* = 26) DOACs (*n* = 26)	Composite of CE, major bleedings, or procedure related major complication	3,4
Zeitler 2023	Observational retrospective study with a PSM 1:1 ratio	Medicare fee-for-service population with a diagnosis of AF (2015–2019), as defined by the chronic conditions warehouse	History of surgical left atrial appendage removal or occlusion	2015–2019	18,776	LAAC (*n* = 9,388) VKA/DOACs (*n* = 9,388)	Mortality, stroke or systemic embolism, and bleeding	1

AF, atrial fibrillation; CE, cardioembolic event; CVD, cardiovascular death; DOACs, direct oral anticoagulants; LAAC, left atrial appendage closure; OAC, oral anticoagulation; PSM, propensity score matching; RCT, randomized control trial; SE, systemic embolism; TIA, transient ischemic attack; VKA, vitamin K antagonist.

**Figure:**
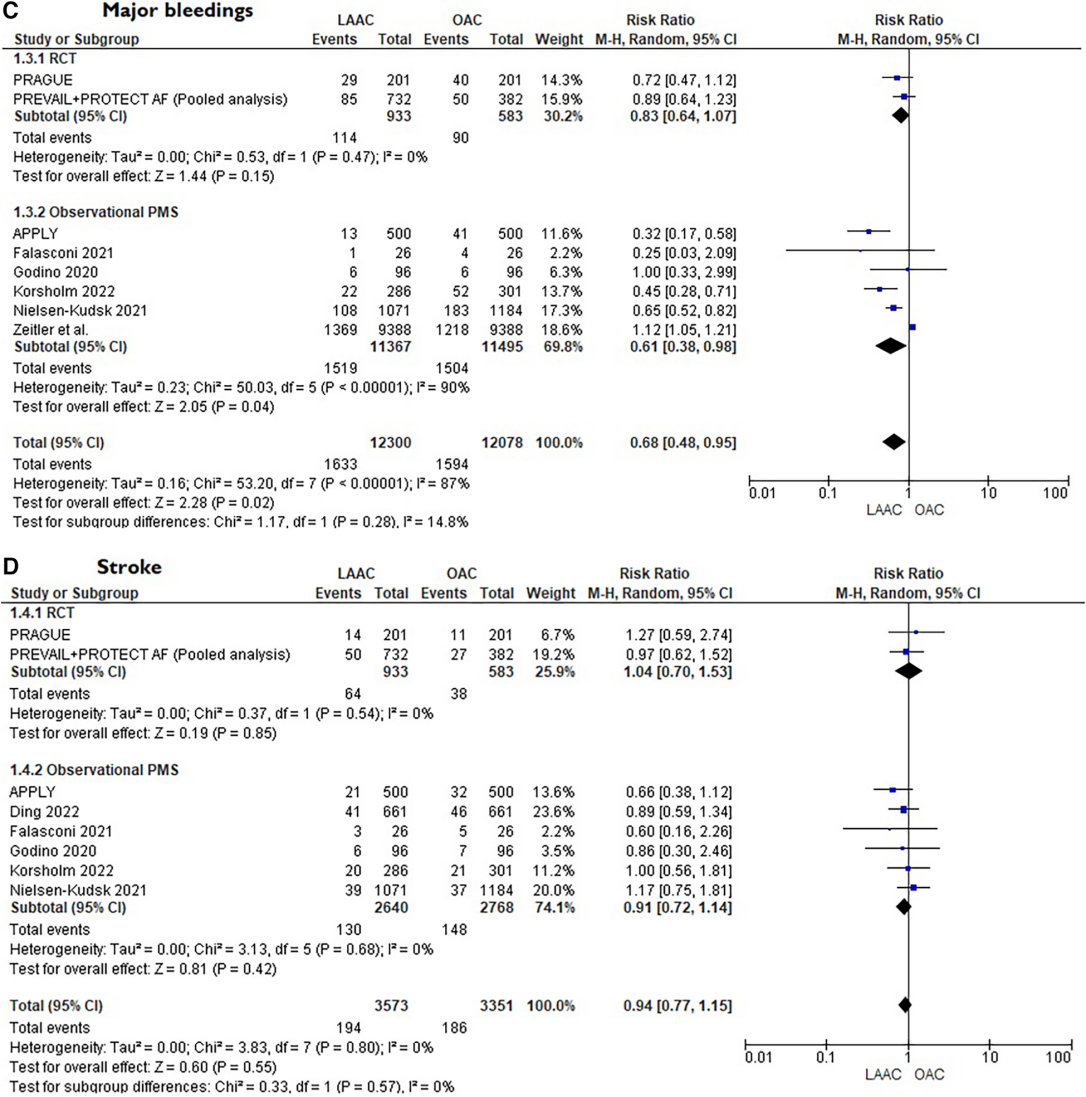


The meta-regression demonstrated no evidence of treatment effect modification by patient characteristics (Age, Sex, HASBLED, VKA, CHA_2_DS_2_-VASc) (see Supplementary Table 1, 2).

According to the Cochrane risk-of-bias tool for RCTs (ROB 2), all the RCTs appraised were classified as low risk for every co-primary outcome appraised (see Supplementary Figures 1–4). ROBINS-I tool (14) demonstrated an overall moderate bias (See Supplementary Tables 3, 4).

## Discussion

To date, the present meta-analysis is the largest evaluating the efficacy and safety of LAAC compared with OAC in AF patients without an absolute contraindication to OAC. The main findings can be summarized as follows:

• LAAC demonstrated a similar efficacy in stroke reduction, compared to OAC, in AF patients.
• Patients after LAAC showed a reduction in all-cause death, CVD and major bleeding compared with OAC.
• No difference was found in the rate of ischemic or hemorrhagic stroke between the two strategies.

In line with previous findings (23, 24), this meta-analysis confirmed that in non-valvular AF patients without an absolute contraindication to OAC, LAAC shows similar efficacy in the prevention of stroke compared with OAC, even at longer follow-up. This result, mainly based on high quality data, is further strengthen by the fact that it maintained consistency both in the leave-one-out sensitivity analysis and in the subgroup analysis according to study design. Moreover, these studies, including patients with a relatively high median CHA2DS2-VASc score, confirmed that LAAC can be considered a safe and feasible therapeutic option even in populations with high-risk profile for systemic embolism. All the RCTs evaluated in this analysis randomized an overall population that could afford long-term OAC. On the other hand, if we consider a real-world cohort of patients with contraindications to OAC, that do not have a therapeutic alternative, the protective role of LAAC might be even greater (25), especially in end-stage renal disease populations where relevant bleeding events were about 10-fold more frequent than stroke or systemic embolism (26). Surprisingly, in our meta-analysis, regardless of stroke type (hemorrhagic or ischemic), no difference was found between the two strategies. Generally, after LAAC implantation, the incidence of ischemic stroke could be even higher compared with medical therapy, and this is mostly due to periprocedural complications or to device malposition that could compromise full long-term protection (27). On the other hand, OAC is usually associated with a major incidence of hemorrhagic stroke that is mostly unraveled by the continuous exposition to the intrinsic bleeding risk carried by OAC. The reason behind this result could be explained by the fact that almost all patients enrolled were on DOACs which, as known, present a safer profile to VKA, especially in elderly populations (28). Subgroup analysis comprising trials with only VKA patients (i.e., PREVAIL + PROTECT AF) (29) further supports this consideration since the risk of hemorrhagic stroke was significantly higher in the OAC arm (see Figure 3B).

The most noteworthy finding of this meta-analysis is the significant reduction of all-cause mortality and CVD in the LAAC group compared with OAC patients. Nonetheless, it must be acknowledged that these findings were mainly driven by the two combined RTCs, (PREVAIL and PROTECT AF) (29) and the largest PMS study (Nielsen-Kudsk et al.) (16). The reason behind the difference in the lower incidence of mortality between this pooled analysis and the PRAGUE 17 may be related to the duration of follow-up. As a matter of fact, in both PROTECT AF and PREVAIL, this net clinical benefit in mortality was not manifest when their results had been published with sensibly shorter follow-up (6, 7). Accordingly, the most convincing hypothesis is that in high bleeding risk individuals, the long-term exposition to OAC, although giving protection from cerebral ischemic events, it exposes to a proportional and progressive increased incidence of serious bleeding complications that may be related to mortality. Therefore, it seems plausible that only the study with the longest follow-up (29) and the one with the largest population appraised (16) were able to show a significant reduction in stronger but rarer endpoints such as death, which usually need longer follow-up to enlighten substantial difference in the treating groups. Moreover, as suggested by previous findings, this net benefit in mortality may be also related to the more favorable neurological outcomes after ischemic cerebrovascular event in LAAC patients rather than patients on OAC (30). Similar conclusions could be made regarding the reduction of major bleedings shown in our metanalysis in the LAAC group. It must be noted that after LAAC, a medical therapy consisting of an antithrombotic regimen of anticoagulation or dual antiplatelet therapy from 1 or 3 to even 6 months (depending on the study protocol) was warranted to protect the patient from periprocedural ischemic complications. The need of this temporary antithrombotic regimen, associated with bleedings related to the intervention, increased initially the risk of major bleedings in the earlier phase of the LAAC strategy, but this risk tended to be progressively counterbalanced in the subsequent months thanks to the cessation of OAC. This aspect is well described in the PRAGUE 17 trial where the superiority of LAAC in reducing the incidence of nonprocedural bleedings became significantly evident only at four years follow-up (31) and not in the early stage (8).

In conclusion, LAAC in non-valvular AF patients is safe and effective in the prevention of stroke and demonstrated a better profile in term of all-cause mortality, CVD and major bleedings compared with OAC, especially after longer follow-up. These findings should stimulate discussion about the role of LAAC as the preferred treatment for the prevention of cardiovascular events in high bleeding risk patients since nowadays, LAAC is often only proposed to patients with an absolute contraindication to OAC (4). However, whether LAAC maintains its protective role throughout life is still unknown. Besides, no standard post-implantation antithrombotic regimen exists to date. Further studies aimed to explore shorter or weaker post LAAC exposure to antithrombotic medication are warranted since might translate into fewer bleeding events, especially during the first 6 months after implantation.

### Limitations

This study has some limitations. Firstly, as inherent to any meta-analysis, this analysis carries the limitations of the original studies. Studies with different OAC strategies were included, considering trials with only VKA patients, DOAC and VKA or just DOAC patients, which may influence the safety and efficacy of results, so a unique interpretation purposely for patients either receiving VKA or a specific DOAC cannot be made. Furthermore, different bleeding definitions were used across all the studies limiting the interpretability of the treatment effect estimates. In older RCTs, LAAC patients were treated early after the procedure with vitamin K antagonist for the first 45 days, then dual antiplatelet therapy for 6 months, and lastly single antiplatelet regimen thereafter, which is a well-known confounding factor in estimating bleeding risk, especially in early days after LAAC. Noteworthy, the antithrombotic regimen on discharge was not standardized throughout the studies and it was tailored mainly according to the clinical characteristics of each patient and the device used potentially affecting the results. Lastly, the relative effect of the device type used in LAAC remains a substantial bias, especially considering the possible differences between WATCHMAN (Boston Scientific, Marlborough, MA, USA) and Amplatzer Cardiac Plug/Amulet device (Abbott, Chicago, IL, USA). Nevertheless, heterogeneity in our study design was very low with scarce variability in patient characteristics variability. At meta-regression analysis, baseline characteristics of the studied cohorts had no impact on the outcomes, finally strengthening the quality of the results.

## Data Availability

The original contributions presented in the study are included in the article/**Supplementary Material**, further inquiries can be directed to the corresponding author.
