# Supplementary material for: Efficacy and safety of left atrial appendage closure compared with oral anticoagulation in atrial fibrillation: a meta-analysis of randomized controlled trials and propensity-matched studies
Source: Front Cardiovasc Med. 2023 Sep 27;10:1212161. doi: 10.3389/fcvm.2023.1212161 (PMC10565038; doi:10.3389/fcvm.2023.1212161)
Supplement: Supplementary file 1 [file Datasheet1.docx]

Supplementary Table 1. Meta-regression analysis for stroke

| VARIABLE | Beta (95%CI) | p |
| --- | --- | --- |
| AGE | -0.002 (-0.12 0.14) | 0.98 |
| FEMALE | -0.007 (-0.07 0.06) | 0.84 |
| CHA_2_DS_2_-VASc | -0,4 (-1,1 0.3) | 0.29 |
| HASBLED | 0.16 (-0.7 1.02) | 0.71 |
| VKA | 0.16 (-0.5 0.83) | 0.63 |

Supplementary Table 2. Meta-regression analysis for major bleedings

| VARIABLE | Beta (95%CI) | p |
| --- | --- | --- |
| AGE | -0.3 (-0.7 0.08) | 0.12 |
| FEMALE | 0.04 (0.015 0.05 | 0.35 |
| CHA_2_DS_2_-VASc | -0.8 (-1.7 0.8) | 0.08 |
| HASBLED | -0.16 (-1.2 0.8) | 0.74 |
| VKA | 0.33 (-0.83 1.49) | 0.58 |

FIGURES

Supplementary Figure 1. Rob2 evaluation for All cause Death

Supplementary Figure 2. ROB2 evaluation for Cardiovascular Death

Supplementary Figure 3. ROB2 evaluation for Stroke

Supplementary Figure 4. ROB2 evaluation for Major Bleedings

|  | Gloekler et al. 2020 (non-RCT) | Godino et al. 2020 (non-RCT) | Nielsen-Kudsk et al. 2021 (non-RCT) |
| --- | --- | --- | --- |
| Bias due to confounding | 1.1 Y ; 1.2 PN ; 1.4 Y ; 1.5 PY ; 1.6 PN ; 1.7 NA Moderate risk | 1.1 Y ; 1.2 PN ; 1.4 Y ; 1.5 PY ; 1.6 PN ; 1.7 NA Moderate risk | 1.1 Y ; 1.2 PN ; 1.4 Y ; 1.5 PY ; 1.6 PN ; 1.7 NA Moderate risk |
| Bias in selection of participants into the study | 2.1 PY ; 2.2 PN ; 2.3 PN ; 2.4 PY ; 2.5 PN  Moderate risk | 2.1 PN ; 2.4 PY ; 2.5 PY  Low risk | 2.1 Y ; 2.2 PY ; 2.3 PY ; 2.4 PN ; 2.5 PN  Moderate risk |
| Bias in classification of interventions | 3.1 Y ; 3.2 PN ; 3.3 PN  Moderate risk | 3.1 Y ; 3.2 Y ; 3.3 PN  Low risk | 3.1 PY ; 3.2 PN ; 3.3 PN  Moderate risk |
| Bias due to deviations from intended interventions | 4.1 PN  Low risk | 4.1 PN  Low risk | 4.1 PN Low risk |
| Bias due to missing data | 5.1 PY ; 5.2 PN ; 5.3 PN  Low risk | 5.1 PY ; 5.2 PN ; 5.3 PN  Low Risk | 5.1 PY Low risk |
| Bias in measurement of outcomes | 6.1 PN ; 6.2 Y ; 6.3 PY ; 6.4 PN  Low risk | 6.1 PN ; 6.2 Y ; 6.3 Y ; 6.4 PN  Low risk | 6.1 PN ; 6.2 Y ; 6.3 PY ; 6.4 PN Low risk |
| Bias in selection of the reported result | 7.1 PN ; 7.2 PN ; 7.3 PN  Low risk | 7.1 PN ; 7.2 PN ; 7.3 PN  Low risk | 7.1 PN ; 7.2 PN ; 7.3 PN Low risk |
| Overall bias | Moderate risk | Moderate risk | Moderate risk |

Supplementary table 3: Risk of bias of individual studies by ROBINS I (non RCTs) guidelines.

Yes (Y), Probably yes (PY), Probably no (PN), No (N), Not appliable (NA), No informations (NI).

|  | Falasconi et al. 2021 (non-RCT) | Ding et al. 2022 (non-RCT) | Korsholm et al. 2022 (non-RCT) | Zeitler et al.  2023 (no-RCT) |
| --- | --- | --- | --- | --- |
| Bias due to confounding | 1.1 Y ; 1.2 PN ; 1.4 Y ; 1.5 PY ; 1.6 PN ; 1.7 NA Moderate risk | 1.1 Y ; 1.2 PN ; 1.4 PY ; 1.5 PY ; 1.6 PN ; 1.7 NA Moderate risk | 1.1 Y ; 1.2 PN ; 1.4 Y ; 1.5 PY ; 1.6 PN ; 1.7 NA Moderate risk | 1.1 Y ; 1.2 PN ; 1.4 PY ; 1.5 PY ; 1.6 PN ; 1.7 NA Moderate risk |
| Bias in selection of participants into the study | 2.1 Y ; 2.2 PY ; 2.3 PN ; 2.4 PY  Moderate risk | 2.1 Y ; 2.2 PN ; 2.4 PY  Moderate risk | 2.1 Y ; 2.2 PY ; 2.3 PY ; 2.4 PN ; 2.5 PN  Moderate risk | .1 Y ; 2.2 PY ; 2.3 PY ; 2.4 PN ; 2.5 PN  Moderate risk |
| Bias in classification of interventions | 3.1 PY ; 3.2 N ; 3.3 PN  Moderate risk | 3.1 PY ; 3.2 N ; 3.3 PN  Moderate risk | 3.1 PY ; 3.2 PN ; 3.3 PN  Moderate risk | 3.1 PY ; 3.2 PN ; 3.3 PN  Moderate risk |
| Bias due to deviations from intended interventions | 4.1 PN  Low risk | 4.1 PN  Low risk | 4.1 PN Low risk | 4.1 PN Low risk |
| Bias due to missing data | 5.1 PY ; 5.2 PN ; 5.3 PN  Low risk | 5.1 PY ; 5.2 PN ; 5.3 PN  Risk | 5.1 PY Low risk | 5.1 PY Low risk |
| Bias in measurement of outcomes | 6.1 PN ; 6.2 Y ; 6.3 PY ; 6.4 PN  Low risk | 6.1 PN ; 6.2 Y ; 6.3 PN ; 6.4 PY  Moderate risk | 6.1 PN ; 6.2 Y ; 6.3 PY ; 6.4 PN Low risk | 6.1 PN ; 6.2 Y ; 6.3 PN ; 6.4 PY  Moderate risk |
| Bias in selection of the reported result | 7.1 PN ; 7.2 PN ; 7.3 PN  Low risk | 7.1 PN ; 7.2 PN ; 7.3 PN  Low risk | 7.1 PN ; 7.2 PN ; 7.3 PN Low risk | 1 PN ; 7.2 PN ; 7.3 PN Low risk |
| Overall bias | Moderate risk | Moderate risk | Moderate risk | Moderate risk |

Supplementary table 4: Risk of bias of individual studies by ROBINS I (non RCTs) guidelines.

Yes (Y), Probably yes (PY), Probably no (PN), No (N), Not appliable (NA), No informations (NI).
